# Supplementary material for: Patterns of Intron Gain and Loss in Fungi
Source: PLoS Biol. 2004 Nov 30;2(12):e422. doi: 10.1371/journal.pbio.0020422 (PMC532390; doi:10.1371/journal.pbio.0020422)
Supplement: Table S1 — Also available at http://genes.mit.edu/NielsenEtAl/. (4.3 MB ZIP). [file pbio.0020422.st001.zip › NielsenEtAl/html/389.html]

AN7674.1.NCU09528.1.MG07449.1.FG05124.1


```
 CLUSTAL W (1.82) Multiple Sequence Alignments - Introns Inserted


Sequence 1: NCU09528.1	232 aa
Sequence 2: FG05124.1	223 aa
Sequence 3: MG07449.1	272 aa
Sequence 4: AN7674.1	248 aa
Alignment Length: 306 aa
Number Identitical Residues: 62 aa
Alignment Score (without introns) 3099


MG07449.1 	MGRTVRQKRKNRSSRPKVKQTNR-----PKKALNPLGNDLIAKNW2NKKETLTQNYRRLG
NCU09528.1	MGREL-QKRKKRSSRAKVQTHTI-----KKKVLNPQGSGIVAKAW2NKKETLSQNYSRFG
FG05124.1 	MGREL-QKKKARSGRQPVRQLNR-----SKKILNPRGNDAIAKSW2NKKETLSQNYRRLG
AN7674.1  	MGNIR-QAKKNRSSAPKQRMKRKGVLKSGKKKINVLGNAIIAENW2DRKATLTQNYRRLG
          	**.   * :* **.    :    .  .: ** :*  *.  :*: * ::* **:*** *:*

MG07449.1 	LLTRLQAPTGGVEPGLRGAADDKSNAHAPLGLS-ERKSTASSAVREVRVKRD-AEGRIVS
NCU09528.1	LVAKLGTAAGGMAKKSAAANYAGITKDDPLAVK-SADRGL-FEVREVKVERD-ASGKIVK
FG05124.1 	LVARLKAPAGGTEKQLGATTTSAYAN-DPFAIA-TIEN---AVVSEARVERD-ADGKIIR
AN7674.1  	LMHRLNAPSGGSEKRKTENGLEEVAS--SLHIKGSVDAMKDSAISETLVERDPKTGKIIR
          	*: :* :.:**                 .: : .  .   .  : *. *:**.  *:*: 

MG07449.1 	VINEE---------ASAAANPLDDPLNELETDS--------------DWEGFGSDDE---
NCU09528.1	VL------------RSD--NPLNDPLNEIESDSESEEPKPKNPTHDIEWHGISDDRQEMI
FG05124.1 	II------------GSAKPNPLNDPLNDLDEGSDAED-KP-----AEEWGGIADNAD---
AN7674.1  	VIRKDDEQVEIAGRKVRSSNPLNDPLNDLSDNE----------------PEIKPQVK---
          	:: ..... . :.     .***:****::. ..                  :  : .   

MG07449.1 	-APTGDRVVDQLERMARVPRVKTVRGLSEREVEWLESLVAKHGDNVKAMERDHRLNPMQQ
NCU09528.1	AQSSRPEVVRMLEEEASRPVEKTVRYQSERELEWLQRLVAKHGDDVAAMVRDIKLNPMQQ
FG05124.1 	-DEETTDVVKQLLEQAKQPDLPKKRHQSAREIEWLEKLVAKYGDDTGAMSRDRKLNPMQQ
AN7674.1  	--KAANQIVQQLEIQADNVAPKKPRHQSKREEEWITRLIEKHGDNYAAMARDRKLNPMQQ
          	       :*  *   *      . *  * ** **:  *: *:**:  ** ** :******

MG07449.1 	TAADISRRLRRLR~G1VWRSLGWIKSKDSSSITVGDRVLELTRCLGFLGVRNEKCQDHPP
NCU09528.1	TKGDITKRLRKAG~-~-------LLQ----------------------------------
FG05124.1 	TSANIAKRLRKMQ~-~-------KESQE--------------------------------
AN7674.1  	TEGDLRRRINKWK0-~-------IYTTIPSQYEVCFLILALWT-----------------
          	* .:: :*:.:                 .:.                             

MG07449.1 	TRAYRRPTV
NCU09528.1	---------
FG05124.1 	---------
AN7674.1  	---------
          	
```
